# Convenience of Hgb-O detected by optical method in XN-series hematology analyzers in evaluating hemoglobin concentration in samples with chylous turbidity

**DOI:** 10.1038/s41598-021-94394-z

**Published:** 2021-07-22

**Authors:** Yu Aruga, Chiaki Ikeda, Arisa Hanai, Sakiko Yoshimura, Momoko Kito, Satoe Miyaki, Misato Tsubokura, Yuka Yasuno, Chiaki Hayashi, Motoi Miyakoshi, Takahiro Nishino, Kimihiko Kawamura, Hiromichi Matsushita

**Affiliations:** Department of Laboratory Medicine, National Cancer Center Hospital, 5-1-1, Tsukiji, Chuo-ku, Tokyo, 104-0045 Japan

**Keywords:** Laboratory techniques and procedures, Diagnostic markers

## Abstract

The chylous turbidity of blood samples is one of the causes of false-high hemoglobin (Hgb) concentration measurements by the colorimetric method, which has been widely applied in hematology analyzers. In such cases, additional manual procedures are required to correct Hgb concentrations. We therefore examined the effectiveness of an optical method for measuring Hgb concentrations in samples with chylous turbidity using Hgb-O in the reticulocyte channel equipped in XN-series analyzers (Sysmex, Kobe, Japan). Hgb-O showed excellent basic performance, including linear correlation and invariability with sodium lauryl sulfate (SLS)-Hgb detected by the colorimetric method. In the analysis of samples from healthy volunteers supplemented with fat emulsion, chylous turbidity did not affect Hgb-O but SLS-Hgb, which was falsely increased according to the dose of fat emulsion. Actually, SLS-Hgb was falsely elevated in 34 of 40 chylous turbidity 3+ samples. The remaining 6 samples were measured in hematology analyzers where Hgb-O was inconsistent with SLS-Hgb in the internal quality control records. For these samples, the correction factors calculated from the internal quality control records could contribute to providing the corrected Hgb-O value. These findings suggested that the optical method was effective and convenient for accurately evaluating Hgb concentrations in samples with extremely chylous turbidity.

## Introduction

The hemoglobin (Hgb) concentration, one of the most important components of complete blood count (CBC), is utilized for evaluating the anemic and polycythemic status of patients. The colorimetric method using hemolysis enables large numbers of samples to be processed in a short time; thus, this method has been widely used in the measurement of Hgb concentrations by hematology analyzers. However, co-existing substances are assumed to cause false-high Hgb concentrations because they affect the color of sample solutions after hemolysis^1–4^.

Chyle, a milky fluid in the blood, is capable of causing analytical interference in biochemical examinations using colorimetric methods, the quantification of serum proteins using immunoturbidimetric assays, and coagulation tests through lipid particles, in addition to falsely decreased electrolyte concentrations due to its volume displacement effect^5–7^. It is also known to be one of the major substances to cause false-high Hgb concentrations^8,9^. It is generated by metabolic abnormalities, including dyslipidemia, hyperlipidemia after meals, and the intravenous administration of fatty emulsion. The degree of chylous turbidity varies, and the correction of measured Hgb concentrations is needed if a blood sample has extremely chylous turbidity. The correction is generally made through the retraction of the supernatant Hgb concentration from the Hgb concentration of whole blood after hemolysis^8,10^ or the measurement of the Hgb concentration after the replacement of chylous plasma by isotonic saline^11^. However, these conventional correction methods require additional manual procedures, such as centrifugal separation, fractionation of supernatant, and replacement of chylous plasma by buffer solution, which may negatively affect the accuracy of laboratory testing.

Many hematology analyzers are equipped with a basic automated operation to evaluate the Hgb concentration using an optical method through the flow cytometric function. Because the optical method requires neither hemolysis nor additional procedures, it may be possible to determine the Hgb concentration with high accuracy in a short time, in case the Hgb concentration measured using the colorimetric method is not secured due to interference by co-existing substances, such as chyle.

The aim of the present study was to validate the effectiveness and convenience of the optical method in the measurement of Hgb concentrations in samples with chylous turbidity.

## Results

### Basic performance of Hgb-O

XN-10 and XN-20 (Sysmex, Kobe, Japan) hematology analyzers were utilized in this study. These devices apply the colorimetric Hgb method using a non-cyanic substrate, sodium lauryl sulfate (SLS-Hgb), as a regular method to qualify the Hgb concentration^12,13^, as well as an optical method of measuring Hgb (Hgb-O) in the reticulocyte (RET) channel, which has not yet been validated in clinical use.

The principle of Hgb-O is as follows: in the RET channel, the CELLPACK DFL™ reagent (Sysmex) generates pores on the cellular membrane of erythrocytes, and the Fluorocell™ RET reagent (Sysmex) stains the inside of erythrocytes. RBC-Y, which is defined as the most frequent value of forward scatter in mature erythrocyte population, is converted to RBC Hgb equivalent (RBC-He), which shows a high correlation with the mean corpuscular hemoglobin (MCH) value. Hgb-O is calculated assuming RBC-He as MCH^14^.

First, the basic performance of Hgb-O was analyzed to validate Hgb-O as a clinical laboratory test.

The correlation with SLS-Hgb in each analyzer (No.1 to No.4, XN-10; No. 5, XN-20) was evaluated using the recorded data from the same patients (n = 1140). The regression equation and correlation coefficient were calculated (Supplementary Fig. S1). The slopes of these equations were 0.998 to 1.070 and all of their correlation coefficients were > 0.980.

The repeatability was evaluated using the coefficient of variation (CV) in 10 consecutive measurements of Hgb-O and SLS-Hgb, which were 0.7 to 1.8% and 0.3 to 0.7%, respectively. Hgb-O showed a larger CV than SLS-Hgb; however, the CV for Hgb-O was still < 2.0% (Supplementary Table S1).

The interference of co-existing substances with the Hgb measurement was evaluated using Interference check A plus (Sysmex). In comparison to SLS-Hgb, the variations (the differences between the maximal and minimal values) of the Hgb-O measurement was smaller in the presence of cell-free Hgb (up to 0.48 g/dL) or chyle (up to 590 FTU), and larger in the presence of indirect or conjugated bilirubin (up to 18.8 mg/dL and 20.2 mg/dL, respectively). In all cases, the variation in the Hgb-O measurement was ≤ 0.3 g/dL (Supplementary Table S2).

These findings demonstrated that Hgb-O had excellent correlation and consistency with SLS-Hgb and that the CVs for the repeatability and variations under the effects of coexisting substrate on Hgb-O measurement were small enough to be negligible. However, the concentrations of triglyceride (TG) and total cholesterol (T-Cho) after the addition of maximal dose (590 FTU) of chyle included in Interference check A plus were 76 mg/dL and 3 mg/dL, respectively, which were much lower in comparison to the TG and T-Cho levels in clinical practice.

### Evaluation of the suitability of Hgb-O for the analysis of samples with chylous turbidity

**Figure 1:**
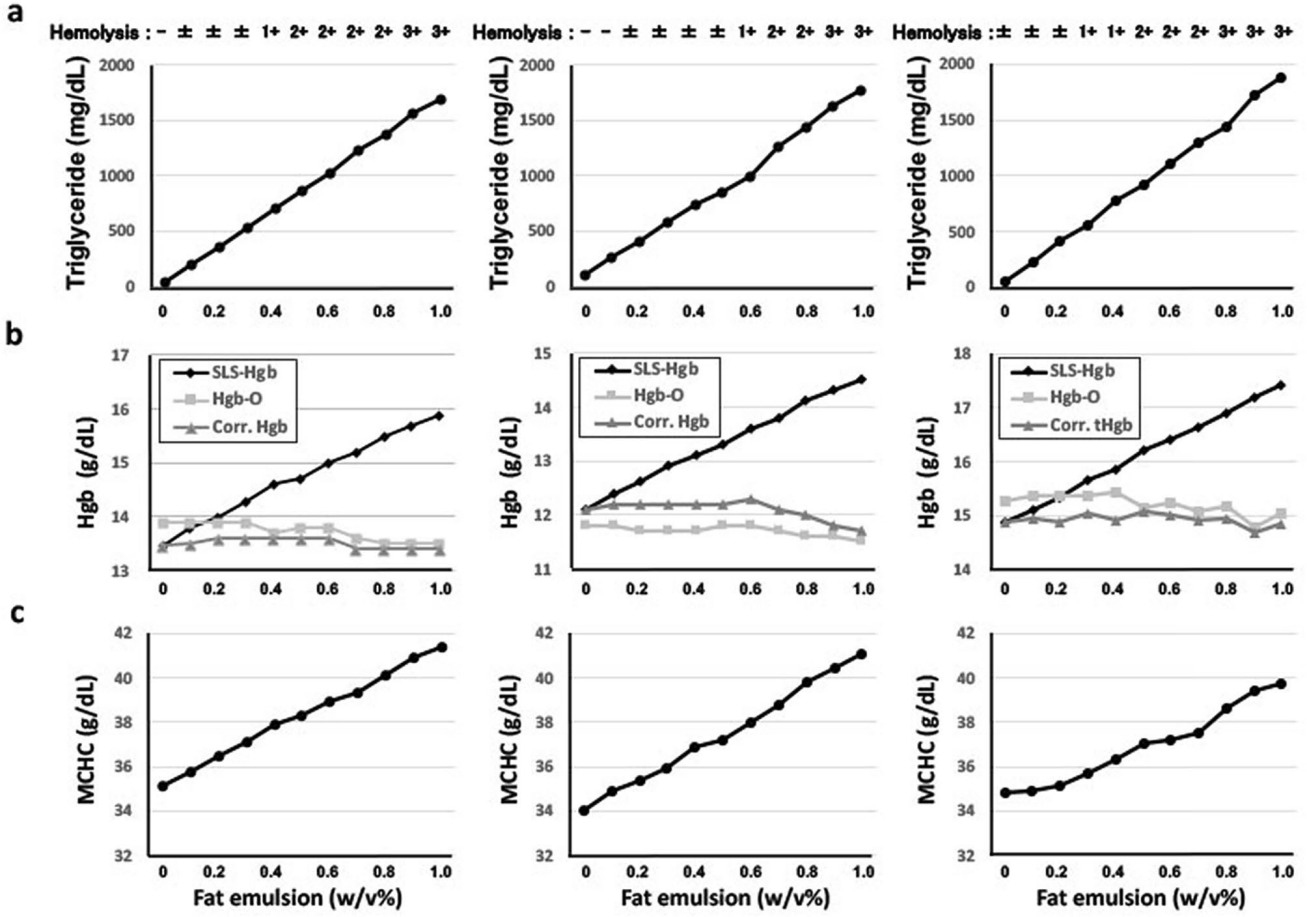
False-high SLS-Hgb values detected in blood samples supplemented with fat emulsion. The blood samples from 3 healthy donors were supplemented with up to 1.0% (w/v) fat emulsion (Intralipos^Ⓡ^). Fat emulsion-dependent changes of (**a**) the serum concentrations of triglyceride, (**b**) SLS-Hgb, Hgb-O and corrected Hgb, and (**c**) MCHC are shown. Corr., Corrected.

In order to evaluate the interference of chylous turbidity in the measurement of Hgb in medical practice, samples supplemented with intravenous fat emulsion, which mainly included TG, were prepared to measure Hgb-O and SLS-Hgb. Increasing the fat emulsion to 1.0% (w/v) in the samples caused the serum appearance to change to a strawberry milk color due to the marked increase in the TG concentration and the resultant hemolysis in the 3 blood samples; the concentrations of serum TG ranged from 42, 114 and 55 mg/dL without fat emulsion to 1694, 1764 and 1885 mg/dL with 1.0% (w/v) fat emulsion, in a dose-dependent manner (Fig. 1a). In contrast, the T-Cho concentration was less markedly affected than the TG concentration in these samples; the T-Cho concentrations ranged from 143 to 162 mg/dL, 170 to 190 mg/dL, and 207 to 198 mg/dL in the 3 blood samples. As expected, the SLS-Hgb level in these samples increased from 13.5 to 15.9 g/dL, 12.1 to 14.5 g/dL, and 14.9 to 17.4 g/dL (Fig. 1b), and the MCHC that was calculated using SLS-Hgb increased accordingly from 35.1 to 41.4 g/dL, 34.0 to 41.1 g/dL, and 34.8 to 39.7 g/dL in each sample (Fig. 1c). The Hgb-O concentration was slightly decreased in all 3 samples, as follows: 13.9 to 13.5 g/dL, 11.8 to 11.5 g/dL and 15.3 to 15.0 g/dL; these changes may reflect hemolysis due to hypertriglyceridemia. However, the Hgb-O data in samples with hypertriglyceridemia were still close to those of corrected Hgb (*see Materials and methods 3. Calculation of corrected Hgb for SLS-Hgb*), suggesting, in contrast to SLS-Hgb, that Hgb-O did not show a false-high value due to the addition of fat emulsion (Fig. 1b).

**Figure 2:**
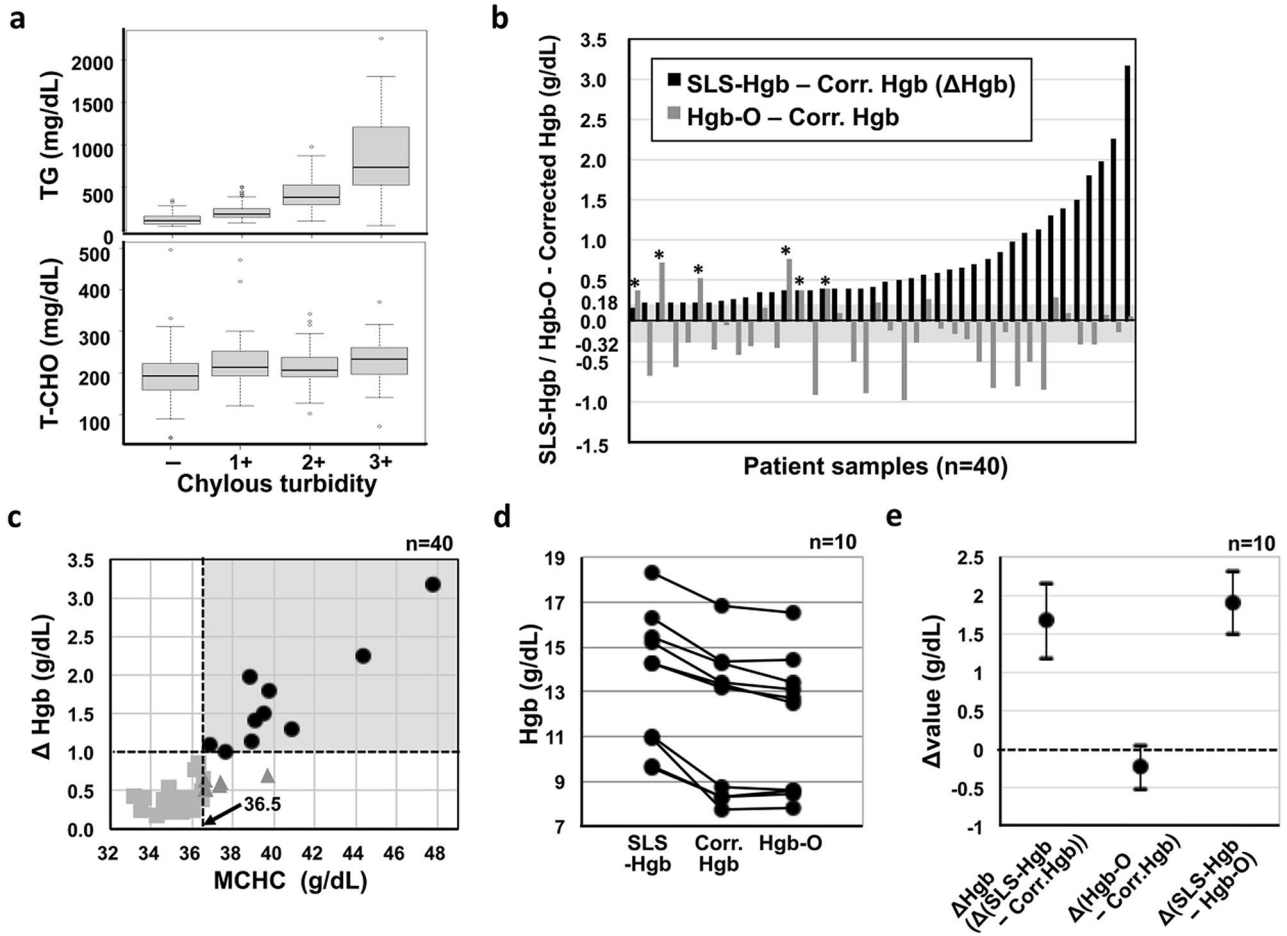
False-high values of SLS-Hgb detected in patient samples. (**a**) The relationship between chylous turbidity and serum lipid levels. (**b**) The values obtained by subtracting corrected Hgb from SLS-Hgb (ΔHgb) or Hgb-O in the 40 chylous turbidity 3+ samples. The shaded area indicates the range of measurement error determined by the repeatability of the intermediate Hgb-O concentration, which was expressed as the widest of the six Hgb datasets shown in Supplementary Table S1. *Denotes samples where Hgb-O was equal to or higher than SLS-Hgb. (**c**) The relationship between ΔHgb and MCHC in the 40 chylous turbidity 3+ samples. The shaded area indicates samples with an MCHC value of > 36.5 g/dL and a ΔHgb value of ≥ 1.0 g/dL. Circles (n = 10) denote the samples with MCHC values of > 36.5 g/dL and ΔHgb values of ≥ 1.0 g/dL, triangles (n = 5) denote samples with MCHC values of > 36.5 g/dL but ΔHgb values of < 1.0 g/dL, and squares (n = 25) denote samples with MCHC values of ≤ 36.5 g/dL and ΔHgb values < 1.0 g/d. (**d**) The comparison of SLS-Hgb, corrected Hgb, and Hgb-O, of the 10 samples in the shaded area. (**e**) The differences in Hgb values (Δvalues) including ΔHgb (SLS-Hgb minus corrected Hgb), Hgb-O minus corrected Hgb, and SLS-Hgb minus Hgb-O. The mean value and 95% CI are shown as representative data. Corr., Corrected.

We then investigated the effect of coexisting chylous turbidity on Hgb measurement using data from patient samples to validate the above-described results. The frequency of chylous turbidity negativity, 1+, 2+ and 3+ in patient samples was 87.4%, 11.6%, 0.9% and 0.1%, respectively. TG increased according to the elevation of chylous turbidity; the median TG values in chylous turbidity negative, 1+, 2+ and 3+ peripheral blood samples were 109 mg/dL, 184 mg/dL, 382 mg/dL and 736 mg/dL, respectively. T-Cho also increased but mildly; the medians of T-Cho in the peripheral blood with chylous turbidity negative, 1+, 2+ and 3+ were 194 mg/dL, 214 mg/dL, 207 mg/dL and 233 mg/dL, respectively (Fig. 2a). Spearman's rank correlation coefficient (ρ) was 0.75 (95% CI: 0.69–0.79) between chylous turbidity and TG whereas it was 0.23 (95% CI: 0.11–0.33) between chylous turbidity and T-Cho, suggesting that chylous turbidity was more strongly correlated with TG than T-Cho, as seen in samples supplemented with fat emulsion.

Using chylous samples (chylous turbidity 1+, n = 17; 2+, n = 21; 3+, n = 40), the differences between SLS-Hgb and corrected Hgb (ΔHgb) were calculated to see whether they were negligible. In the chylous turbidity 1+ and 2+ samples, the ΔHgb value ranged from 0.0 to 0.2 g/dL (median 0.1 g/dL). However, the ΔHgb value ranged from 0.2 to 3.2 g/dL (median 0.4 g/dL) in the chylous turbidity 3+ samples, which was not negligible. The 40 turbidity 3+ samples showed a high frequency of hemolysis (2+, n = 5, 12.5%; 1+, n = 10, 25.0%; +/−, n = 22, 55.0%) (Supplementary Table S3), indicating a sharp contrast to the frequency of hemolysis samples: 3+, 0.0%; 2+, 0.1%; 1+, 0.2%; +/−, 1.6%; negativity, 98.1%. On the other hand, Hgb-O was close to the corrected Hgb value and was lower than SLS-Hgb in 34 of the 40 chylous turbidity 3+ samples (Fig. 2b, Supplementary Table S3). Additionally, MCHC was higher than 36.5 g/dL, which was defined as the lower limit of high MCHC values in a previous study^9^, in 15 of the 40 chylous turbidity 3+ samples, and 10 of these 15 samples showed ΔHgb values of ≥ 1.0 g/dL (Fig. 2c). In all 10 samples, Hgb-O was equal to the corrected Hgb value and was apparently lower than SLS-Hgb (Fig. 2d), which were supported by the 3 mean differences in Hgb values (Δvalues), including ΔHgb, Hgb-O minus corrected Hgb, and SLS-Hgb minus Hgb-O (Fig. 2e). In contrast, there were no samples in which the ΔHgb was ≥ 1.0 g/dL when MCHC was ≤ 36.5 g/dL.

These experimental and clinical observation clearly revealed that TG was the main component of chyle that affected SLS-Hgb but not Hgb-O. It is therefore recommended that Hgb-O be utilized for the measurement of Hgb when a sample is chylous turbidity 3+ with an MCHC value of > 36.5 g/dL.

### Investigation of the quality of Hgb-O in hematology analyzers

As shown in Fig. 2b, Hgb-O was equal to or higher than SLS-Hgb in 6 of the 40 chylous turbidity 3+ samples. These results were inconsistent with the experimental data shown in Fig. 1b. The fact that an internal quality control system for Hgb-O has not been established may be one of the reasons for the inconsistency.

**Figure 3:**
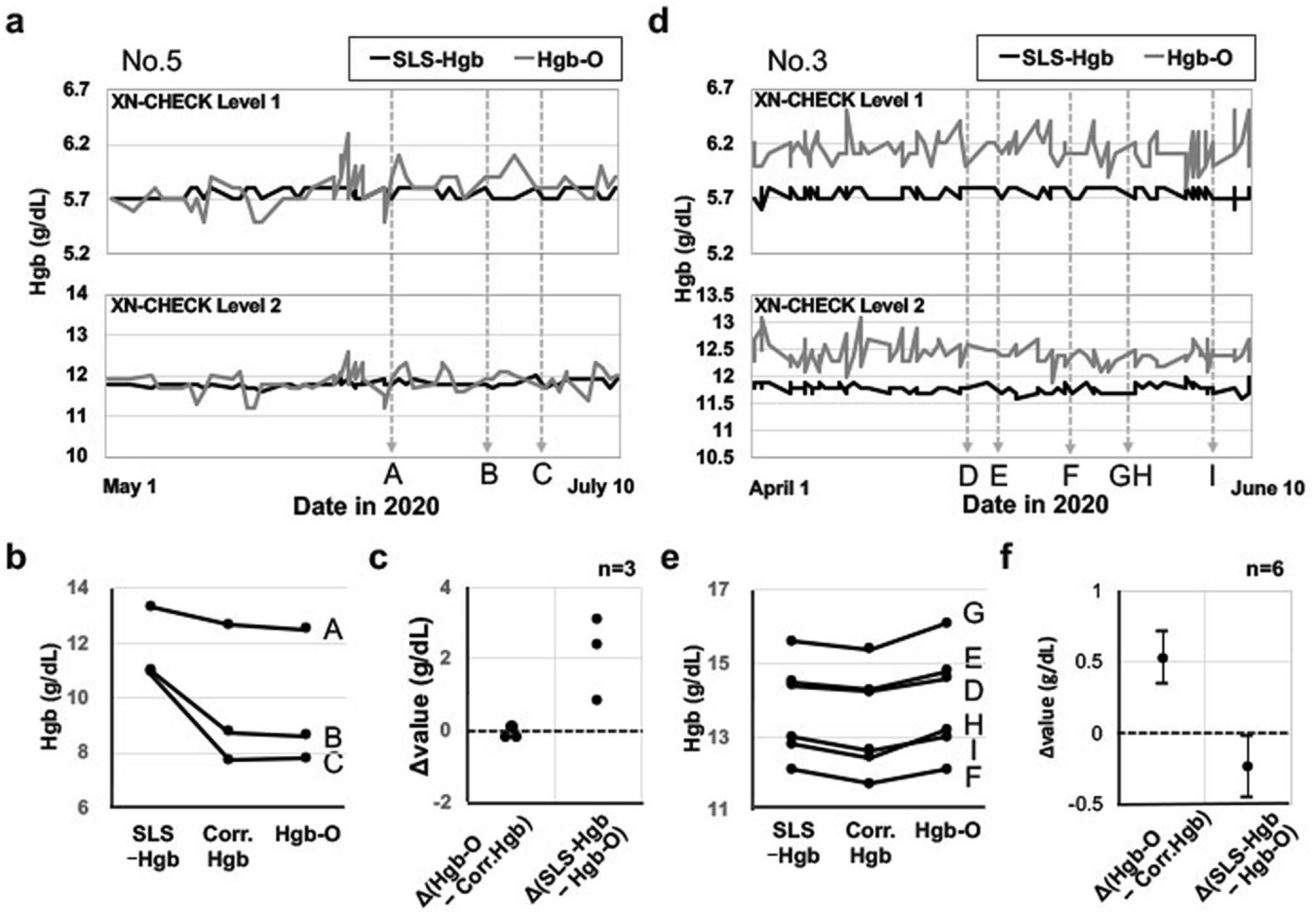
The time-dependent changes of Hgb-O in comparison to quality-controlled SLS-Hgb measured using XN-CHECK Level 1 and 2. (**a**) The No. 5 hematology analyzer showed good correlation between Hgb-O and SLS-Hgb. The data of May 1st to July 10th from the 1-year observation period from June 2019 to July 2020 are depicted. “A” to “C” denote the time-points at which the samples shown in (**b**) were measured. (**b**) The comparison of SLS-Hgb, corrected Hgb, and Hgb-O of the 3 samples (A to C) analyzed by the No. 5 hematology analyzer in the period shown in (**a**). (**c**) The differences in Hgb values (Δvalues), including Hgb-O minus corrected Hgb and SLS-Hgb minus Hgb-O. Each dot represents each sample. (**d**) The No. 3 hematology analyzer showed the constant elevation of Hgb-O. The data of April 1st to June 10th from the 1-year observation period from June 2019 to July 2020 are depicted. “D” to “I” denote the time-points at which the samples shown in (**e**) were measured. (**e**) The comparison of SLS-Hgb, corrected Hgb, and Hgb-O of the 6 samples (D to I) analyzed by the No. 3 hematology analyzer in the period shown in (**d**). (**f**) The differences in Hgb values (Δvalues), including Hgb-O minus corrected Hgb and SLS-Hgb minus Hgb-O. The mean values with 95% confidence intervals are shown. Corr., Corrected.

In order to investigate the quality status of Hgb-O, the internal quality control records of SLS-Hgb and the corresponding Hgb-O data in each hematology analyzer were compared. First, the regression equations comparing the data from No. 2 to 5 with the reference data from No.1 were calculated. The slopes of the regression equations of SLS-Hgb converged on 0.99 to 1.01, but those of Hgb-O ranged from 0.97 to 1.09. Then, we investigated the time-dependent changes in Hgb-O in comparison to quality-controlled SLS-Hgb using XN-CHECK control blood (Sysmex, Kobe, Japan) in each hematology analyzer between June 2019 and July 2020. There were discrepancies between Hgb-O and SLS-Hgb, and they varied according to the hematology analyzers that were used. The No. 5 hematology analyzer showed good correlation between Hgb-O and SLS-Hgb during the study period (Fig. 3a), and the slope of the regression equation using 100 patient samples was 1.030 (Supplementary Fig. S2). In 3 chylous turbidity 3+ samples that were measured by the No. 5 hematology analyzer in the study period, Hgb-O was almost equal to the corrected Hgb value, and was always lower than SLS-Hgb (Fig. 3b, c). On the other hand, the No. 3 hematology analyzer showed the constant elevation of Hgb-O in the XN-CHECK Level 1 and 2 measurements (Fig. 3d), therefore the resulting elevation of the slope of the regression equation was 1.098 (Supplementary Fig. S2). Hgb-O measured by the No. 3 hematology analyzer in the period were equal (n = 2) or higher (n = 4) in comparison to SLS-Hgb in 6 chylous turbidity 3+ samples (Fig. 3e). This was consistent with the positive value of the mean difference in Hgb-O minus corrected Hgb and the negative value of the mean difference in SLS-Hgb minus Hgb-O (Fig. 3f).

These findings demonstrate that Hgb-O showed variable discrepancy from SLS-Hgb because the quality of Hgb-O was not controlled. It is therefore important to check the internal quality control records of SLS-Hgb data and the corresponding Hgb-O data before evaluating Hgb-O, in order to see the degree of discrepancy between them.

### Correction of Hgb-O using the internal quality control records

**Figure 4:**
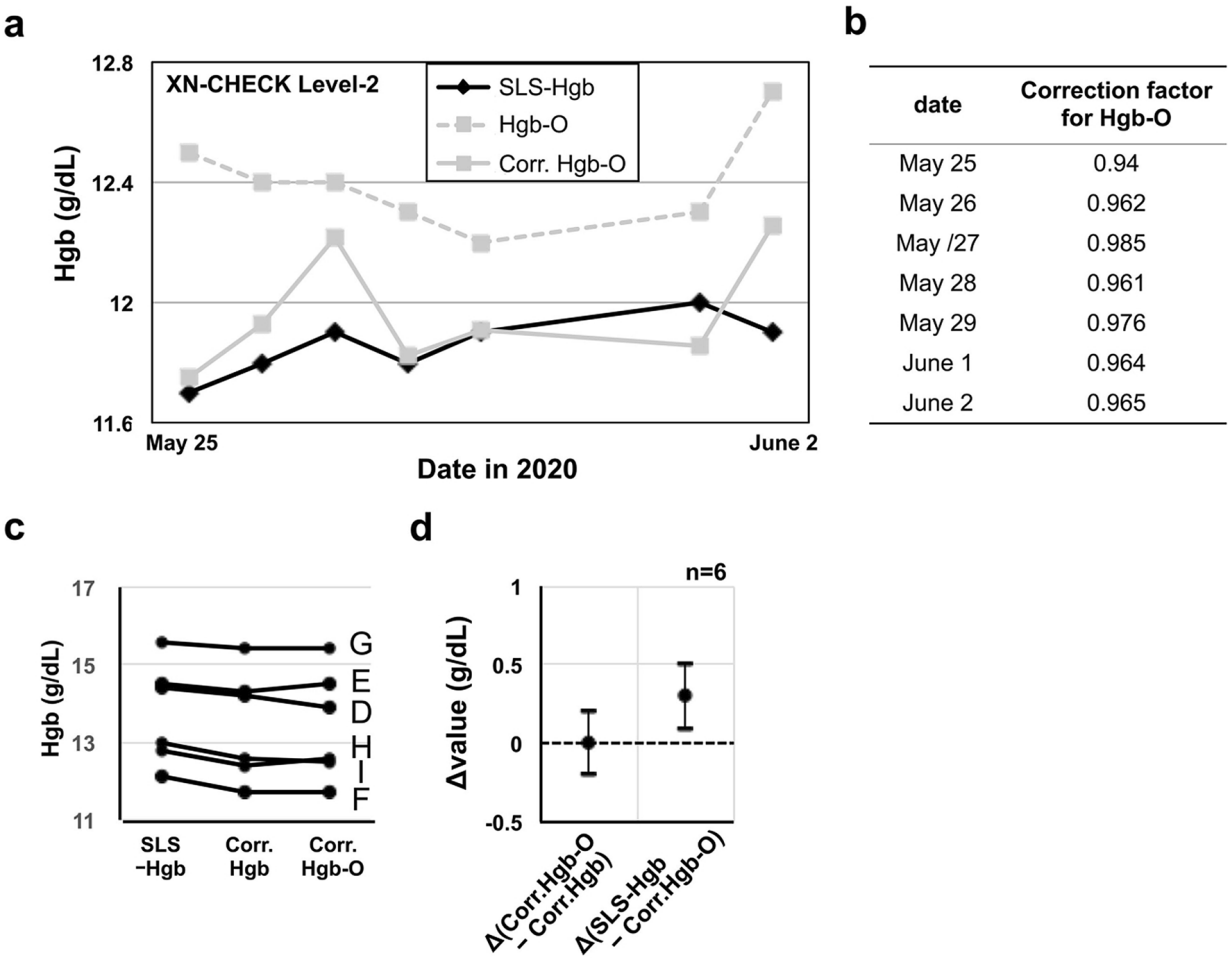
The correction of Hgb-O by the correction factor. (**a**) The time-dependent changes of quality-controlled SLS-Hgb, Hgb-O and corrected Hgb-O in the No. 3 hematology analyzer. The corrected Hgb-O was calculated using the correction factor for Hgb-O, which is shown in (**b**). (**c**) The comparison of SLS-Hgb, corrected Hgb and corrected Hgb-O, instead of Hgb-O, of the 6 samples (D to I) as shown in Fig. 3d. (**d**) The differences in Hgb values (Δvalues) including corrected Hgb-O minus corrected Hgb and SLS-Hgb minus corrected Hgb-O. The mean values with 95% CI are shown. Corr., Corrected.

To overcome discrepancy between the methods, the validity of the correction factors for Hgb-O were verified. The correction factor represented the proportion of the mean SLS-Hgb value to the mean Hgb-O value, which was calculated from the daily quality control data using 5 patient samples in each hematology analyzer. The measured Hgb-O values were multiplied by the corresponding correction factor to obtain the corrected Hgb-O value. The discrepancy between SLS-Hgb and Hgb-O in the dairy internal quality control became inconspicuous when Hgb-O was substituted for corrected Hgb-O (Fig. 4a, b). Additionally, all of the corrected Hgb-O calculated from the 6 measured Hgb-O values that were equal to or higher than SLS-Hgb (Figs. 2b, 3f) were almost equal to corrected Hgb, and were equal to (n = 1) or lower than SLS-Hgb (Fig. 4c). In these 6 samples, the mean difference in corrected Hgb-O minus corrected Hgb was almost 0, and the mean difference in SLS-Hgb minus corrected Hgb-O was positive (Fig. 4d).

These findings suggested that the calculation of corrected Hgb-O using the correction factors is reasonable for the evaluation of the Hgb concentration in cases where there is a discrepancy between SLS-Hgb and Hgb-O.

## Discussion

The current study demonstrates the effectiveness of Hgb-O measured in the RET channel of the XN-10 and XN-20 hematology analyzers using samples with chylous turbidity; Hgb-O not only had good correlation with SLS-Hgb, but also exhibited values that were close to the corrected Hgb value even in the measurement of samples with chylous turbidity where the TG concentration were mainly elevated. An MCHC value of > 36.5 g/dL was a good parameter to select samples with chylous turbidity 3+ for the application of Hgb-O. Among these samples, the frequency of samples showing ≥ 1.0 g/dL of difference between SLS-Hgb and Hgb-O was high. However, there were samples where Hgb-O data required adjustment (shown as corrected Hgb-O), because of the lack of a quality control system.

The colorimetric method detects both cellular and cell-free Hgb without distinguishing between them. Substances, such as cell-free Hgb, bilirubin and chyle, may interfere with measurements and cause false-high Hgb values when using the colorimetric method^1–4^. In particular, severe chylous turbidity with a high TG concentration has been reported to induce hemolysis^15,16^, which was also seen in the artificially generated samples supplemented with intravenous fat emulsion (Fig. 1b) and in the clinical samples (Supplementary Table S3), exacerbating the false-high SLS-Hgb value caused by chyle. It is obvious that the most beneficial advantage of optical methods, such as Hgb-O, is that they are able to selectively detect cellular hemoglobin. Notably, the present study revealed that Hgb-O was scarcely affected by 18.8 mg/dL of indirect bilirubin and 20.2 mg/dL of conjugated bilirubin in the present study, although SLS-Hgb also showed an insusceptibility to co-existing bilirubin (Supplementary Table S2). Therefore, Hgb-O measurement is effective for samples with severe chylous turbidity, which interrupted the SLS-Hgb measurement due to a false-high value.

Although hematology analyzers from various manufactures are often equipped with a system for detecting cellular hemoglobin, a limited number of reports related to the optical method of Hgb measurement have been published. Kunicka et al. reported that the optical method showed good correlation with the colorimetric method when using the ADVIA 120 system (Siemens)^17^. Berda-Haddad et al. suggested that Hgb-O was one of the recommended tools for the evaluation of the Hgb concentration in samples with MCHC values of > 36.5 g/dL^9^. Gönel et al. reported that cellular Hgb measured by the optical method using the CELLDYN Ruby System (Abbott, Lake Forest, USA) was effective for obtaining the correct Hgb value in an excessively lipemic sample^18^. However, the former study did not show the effectiveness of the colorimetric method for the analysis of chylous samples, and the latter two only included a small number of chylous samples. Thus, the current study is the first report to clearly describe the convenience of the optical method for the measurement of Hgb in chylous samples with detailed analyses for practical use.

MCHC, an important parameter for chylous samples, is also elevated under several conditions, including hyposmolar plasma conditions and under conditions of erythroid diseases, such as intravascular hemolysis, erythrocyte agglutination, hereditary spherocytosis and sickle cell anemia. The upper limit of the reference range of MCHC is generally defined as 36.0 g/dL, and MCHC values of > 36.0 g/dL are rarely observed^19–21^. Actually, the frequency of MCHC values of > 36.0 g/dL and > 36.5 g/dL in 959,397 samples obtained in our hospital was as low as 1.12% and 0.41%, respectively (unpublished data). High MCHC values are not specific to chylous turbidity and the cause requires discrimination; however, they are effective for initial screening to identify samples with chylous turbidity 3+, which should be subjected to an Hgb-O analysis. In particular, MCHC is more useful as a parameter for Hgb-O analyses than chylous turbidity, as both MCHC and Hgb-O can be easily measured with the same analyzer.

One important issue is that there is currently no established system for managing quality control for Hgb-O. Calibrators have been identified that correspond specifically to leukocyte differentials, RET, PLT-O and PLT-F in XN-10 and XN-20, which are the parameters used in the optical method, but not for RBC-Y and RBC-He, which are used in the calculation of Hgb-O. In addition, the wider fluctuation of the Hgb-O level using XN-CHECK than quality-controlled SLS-Hgb suggests that Hgb-O is less stable than SLS-Hgb (Fig. 3a,d). Because the frequency of chylous (3+) and/or an MCHC value of > 36.5 g/dL is very low in clinical samples, it is reasonable and appropriate to measure the Hgb-O values only in these limited samples, and thus it is not necessary to apply it for all the samples. At the moment, it is important to confirm that there is no discrepancy in the quality management between SLS-Hgb and Hgb-O, and the correction factor should be applied if there is an undeniable discrepancy between them.

In conclusion, the current study validated the effectiveness and convenience of the optical method of Hgb measurement in samples with chylous turbidity 3+ in comparison to the colorimetric method. Applying Hgb-O in practical use will help to provide accurate Hgb measurements, and shed light on false-high Hgb values, which have been uncovered using the colorimetric method. Because this study performed was in a single cancer center hospital, the frequency and thus the total number of samples with chylous turbidity 3+ might be much higher in comparison to general hospitals and clinics. It is therefore strongly expected that the optical method of Hgb measurement, with an adequate quality control system, be widely applied in the near future in order to facilitate the accurate measurement of the Hgb concentration in samples with chylous turbidity.

## Materials and methods

### Evaluation of the basic performance of Hgb-O

The correlation between Hgb-O and SLS-Hgb, including the regression equation and correlation coefficient was evaluated using the hematological data of patient samples recorded in the XN-10 and XN-20 hematology analyzers between June 2019 to June 2020 (n = 1140) for internal quality control.

Repeatability was evaluated by the coefficient of variation (CV), which was calculated by 10-times measurement in 3 patient samples with different Hgb concentrations (low, 1; intermediate, 1; high, 1).

The interference of coexisting substances was evaluated based on the changes in the mean values of triplicate measurements after the addition of various concentrations of substances (cell-free Hgb, indirect or conjugated bilirubin, and chyle) included in the Interference check A plus (Sysmex).

### Interference of chylous turbidity in Hgb measurement using artificially generated samples

Nine volumes of blood samples obtained from 3 healthy volunteers were mixed with one volume of saline, which was serially-diluted with intravenous fat emulsion Intralipos^Ⓡ^ Injection10% (Otsuka Pharmaceutical, Tokyo, Japan) to generate blood samples including fat emulsion at a final concentration of 0 to 1.0% (w/v). SLS-Hgb and Hgb-O were measured in triplicate, and the mean values were calculated.

### Calculation of corrected Hgb for SLS-Hgb

The corrected Hgb value was calculated using the following formula: Corrected Hgb = SLS-Hgb(g/dL) − (1-Hematocrit) × cell-free Hgb (g/dL)^8,10^.

The cell-free Hgb was the Hgb concentration in the plasma supernatant which was measured using an XN-10 or XN-20 hematology analyzers after centrifugation (3000 rpm for 5 min).

### Biochemical analyzer and chylous index

Biochemical analyses, such as the measurement of TG and T-Cho in the serum and plasma, were performed using a LABOSPECT 008 (Hitachi High-Technologies, Tokyo, Japan). The analyzer could provide the chylous index, which expressed cloudiness due to chyle, to evaluate sample quality. This was calculated by dividing the absorbance at a wavelength of 660 to 700 nm by 320. Our institute defined chylous index 1, 2 to 3, and ≥ 4 as chylous turbidity 1+, 2+, and 3+, respectively.

### Investigation of the interference by chylous turbidity in Hgb measurements and the frequency of hemolysis using clinical records

The frequency of chylous and hemolytic samples, the relationship between chylous index and blood lipid concentrations (TG and T-Cho), the comparison of Hgb-O, SLS-Hgb and corrected Hgb in the 40 chylous 3+ samples, the frequency of high MCHC values measured in our clinical laboratory were evaluated using clinical records from April 2019 to March 2020, April 2019 to March 2020, May 2018 to August 2020, April 2014 to June 2019, respectively.

### Investigation of the quality of Hgb-O in hematology analyzers

The internal quality control records of SLS-Hgb, as measured using an XN-CHECK control blood (Sysmex) and patient samples from April 2019 to July 2020, and the quality records of Hgb-O in the corresponding period were investigated and compared.

### Statistical analysis

All statistical analyses were performed using R version 4.0.3. A linear regression analysis and Pearson’s correlation coefficient (r) were used for comparison between two measurements or hematology analyzers. Spearman's rank correlation coefficients (ρ) were calculated to evaluate the correlation between chylous turbidity and triglyceride or total cholesterol. The mean values of differences (Δvalue) between two different Hgb measurement methods, including SLS-Hgb, Hgb-O, corrected Hgb and corrected Hgb-O, were calculated with the 95% confidence interval (95% CI).

### Ethical approval

This study was conducted in accordance with the principles of the Declaration of Helsinki and was approved by the Institutional Review Board of the National Cancer Center (#2016-403 and #2020-125). Written informed consent was obtained from the healthy donors and the patients.

## Supplementary Information

Supplementary Information.
