# Supplementary material for: Convenience of Hgb-O detected by optical method in XN-series hematology analyzers in evaluating hemoglobin concentration in samples with chylous turbidity
Source: Sci Rep. 2021 Jul 22;11:14978. doi: 10.1038/s41598-021-94394-z (PMC8298502; doi:10.1038/s41598-021-94394-z)

**Convenience of Hgb-O detected by optical method in XN-series hematology analyzers in evaluating hemoglobin concentration in samples with chylous turbidity**

Yu Aruga1, Chiaki Ikeda1, Arisa Hanai1, Sakiko Yoshimura1, Momoko Kito1, Satoe Miyaki1,

Misato Tsubokura1, Yuka Yasuno1, Chiaki Hayashi1, Motoi Miyakoshi1, Takahiro Nishino1,

Kimihiko Kawamura1, and Hiromichi Matsushita1

^1^Department of Laboratory Medicine, National Cancer Center Hospital, 5-1-1, Tsukiji, Chuo-ku,

Tokyo 104-0045, Japan

Supplementary Table S1.

The repeatability of Hgb-O.

|  | Hgb（low） | | Hgb（intermediate） | | Hgb（high） | |
| --- | --- | --- | --- | --- | --- | --- |
|  | SLS-Hgb | Hgb-O | SLS-Hgb | Hgb-O | SLS-Hgb | Hgb-O |
| Minimum (g/dL) | 6.8 | 6.5 | 15.4 | 15.2 | 17.5 | 17.5 |
| Maximum (g/dL) | 6.9 | 6.9 | 15.5 | 15.7 | 17.9 | 17.9 |
| Mean (g/dL) | 6.86 | 6.77 | 15.44 | 15.52 | 17.77 | 17.72 |
| SD (g/dL) | 0.05 | 0.12 | 0.05 | 0.15 | 0.13 | 0.13 |
| CV (%) | 0.7 | 1.8 | 0.3 | 1.0 | 0.7 | 0.7 |

CV, coefficient of variation; SD, standard deviation

Supplementary Table S2.

The interference of co-existing substances on the Hgb measurement.

| Cell-free Hgb (mg/dL) | SLS-Hgb (g/dL) | Hgb-O (g/dL) | Indirect bilirubin (mg/dL) | SLS-Hgb (g/dL) | Hgb-O (g/dL) | Conjugated bilirubin (mg/dL) | SLS-Hgb (g/dL) | Hgb-O (g/dL) | Chyle (FTU) | SLS-Hgb (g/dL) | Hgb-O (g/dL) |
| --- | --- | --- | --- | --- | --- | --- | --- | --- | --- | --- | --- |
| 0 | 14.3 | 13.9 | 0 | 13.9 | 13.2 | 0 | 13.3 | 13.1 | 0 | 13.6 | 13.1 |
| 96 | 14.4 | 14.0 | 3.8 | 13.9 | 13.5 | 4 | 13.4 | 13.3 | 318 | 13.7 | 13.0 |
| 288 | 14.6 | 14.1 | 11.3 | 14.0 | 13.4 | 12.1 | 13.5 | 13.2 | 954 | 14.0 | 13.2 |
| 480 | 14.7 | 14.0 | 18.8 | 14.0 | 13.3 | 20.2 | 13.4 | 13.0 | 1590 | 13.9 | 13.0 |
| Minimum  (Min) | 14.3 | 13.9 | Minimum  (Min) | 13.9 | 13.2 | Minimum  (Min) | 13.3 | 13.0 | Minimum  (Min) | 13.6 | 13.0 |
| Maximum  (Max) | 14.7 | 14.1 | Maximum  (Max) | 14.0 | 13.5 | Maximum  (Max) | 13.4 | 13.3 | Maximum  (Max) | 14.0 | 13.2 |
| Variation  (Max - Min) | 0.4 | 0.2 | Variation  (Max - Min) | 0.1 | 0.3 | Variation  (Max - Min) | 0.1 | 0.3 | Variation  (Max - Min) | 0.4 | 0.2 |

Supplementary Table S3.

Hemoglobin concentration and the related data in the 40 chylous turbidity 3+ samples.

| Sample No. | SLS-Hgb（g/dL） | Corrected Hgb (g/dL) | *ΔHgb (g/dL) | Hgb-O (g/dL) | MCHC (g/dL) | Cell-free Hgb (g/dL) | Hematocrit | RBC (×10^4^/µL) | Hemolysis |
| --- | --- | --- | --- | --- | --- | --- | --- | --- | --- |
| 1 | 10.9 | 7.7 | 3.2 | 7.8 | 47.8 | 4.1 | 0.228 | 274 | 1+ |
| 2 | 11.0 | 8.7 | 2.3 | 8.6 | 44.4 | 3.0 | 0.248 | 292 | 1+ |
| 3 | 9.6 | 8.3 | 1.3 | 8.6 | 40.9 | 1.7 | 0.235 | 286 | 1+ |
| 4 | 15.2 | 13.4 | 1.8 | 13.1 | 39.8 | 2.9 | 0.382 | 397 | 2+ |
| 5 | 10.0 | 9.3 | 0.7 | 8.8 | 39.7 | 0.9 | 0.252 | 291 | +/- |
| 6 | 18.3 | 16.8 | 1.5 | 16.5 | 39.5 | 2.8 | 0.463 | 511 | 2+ |
| 7 | 9.7 | 8.3 | 1.4 | 8.4 | 39.1 | 1.9 | 0.248 | 270 | +/- |
| 8 | 15.4 | 14.3 | 1.1 | 13.4 | 38.9 | 1.9 | 0.396 | 418 | 1+ |
| 9 | 16.3 | 14.3 | 2.0 | 14.4 | 38.8 | 3.4 | 0.420 | 441 | 2+ |
| 10 | 14.3 | 13.3 | 1.0 | 12.5 | 37.6 | 1.6 | 0.380 | 375 | 1+ |
| 11 | 9.2 | 8.6 | 0.6 | 8.5 | 37.4 | 0.8 | 0.246 | 269 | +/- |
| 12 | 13.7 | 13.1 | 0.6 | 13.4 | 37.3 | 0.9 | 0.367 | 387 | 1+ |
| 13 | 14.3 | 13.2 | 1.1 | 12.7 | 36.9 | 1.8 | 0.388 | 397 | 2+ |
| 14 | 13.3 | 12.7 | 0.6 | 12.5 | 36.6 | 1.0 | 0.363 | 383 | 1+ |
| 15 | 13.4 | 12.9 | 0.5 | 11.9 | 36.6 | 0.8 | 0.366 | 361 | +/- |
| 16 | 14.5 | 13.8 | 0.7 | 13.6 | 36.5 | 1.1 | 0.397 | 379 | 1+ |
| 17 | 18.8 | 18.3 | 0.5 | 18.2 | 36.5 | 1.0 | 0.515 | 500 | 1+ |
| 18 | 13.0 | 12.6 | 0.4 | 11.7 | 36.5 | 0.6 | 0.356 | 310 | +/- |
| 19 | 17.9 | 17.0 | 0.9 | 16.9 | 36.3 | 1.7 | 0.493 | 543 | 2+ |
| 20 | 14.6 | 13.8 | 0.8 | 13.0 | 36.1 | 1.3 | 0.404 | 422 | +/- |
| 21 | 15.5 | 15.2 | 0.3 | 14.9 | 36.0 | 0.5 | 0.431 | 454 | +/- |
| 22 | 18.2 | 18.0 | 0.2 | 17.9 | 36.0 | 0.5 | 0.505 | 494 | 1+ |
| 23 | 13.0 | 12.6 | 0.4 | 13.0 | 35.8 | 0.6 | 0.363 | 378 | +/- |
| 24 | 15.1 | 14.7 | 0.4 | 13.8 | 35.7 | 0.7 | 0.423 | 472 | +/- |
| 25 | 14.3 | 13.9 | 0.4 | 14.1 | 35.7 | 0.6 | 0.401 | 422 | +/- |
| 26 | 11.3 | 11.0 | 0.3 | 10.6 | 35.5 | 0.4 | 0.318 | 318 | - |
| 27 | 16.1 | 15.9 | 0.2 | 15.2 | 35.5 | 0.4 | 0.454 | 488 | +/- |
| 28 | 15.0 | 14.8 | 0.2 | 14.5 | 35.5 | 0.4 | 0.422 | 479 | +/- |
| 29 | 15.8 | 15.6 | 0.2 | 15.0 | 35.3 | 0.4 | 0.447 | 478 | +/- |
| 30 | 15.1 | 14.7 | 0.4 | 14.8 | 35.2 | 0.7 | 0.429 | 454 | +/- |
| 31 | 12.1 | 11.7 | 0.4 | 12.1 | 35.0 | 0.6 | 0.346 | 351 | +/- |
| 32 | 14.2 | 13.7 | 0.5 | 13.4 | 34.9 | 0.9 | 0.407 | 438 | +/- |
| 33 | 13.8 | 13.4 | 0.4 | 13.1 | 34.9 | 0.6 | 0.395 | 379 | +/- |
| 34 | 14.5 | 14.3 | 0.2 | 14.8 | 34.9 | 0.4 | 0.415 | 426 | +/- |
| 35 | 15.6 | 15.4 | 0.2 | 16.1 | 34.7 | 0.4 | 0.450 | 467 | - |
| 36 | 12.8 | 12.4 | 0.4 | 13.2 | 34.6 | 0.6 | 0.370 | 412 | +/- |
| 37 | 14.4 | 14.2 | 0.2 | 14.6 | 34.3 | 0.3 | 0.420 | 399 | - |
| 38 | 14.4 | 14.0 | 0.4 | 13.5 | 33.6 | 0.7 | 0.428 | 428 | +/- |
| 39 | 13.8 | 13.6 | 0.2 | 13.2 | 33.5 | 0.4 | 0.412 | 416 | +/- |
| 40 | 12.9 | 12.5 | 0.4 | 12.7 | 33.2 | 0.7 | 0.389 | 435 | +/- |

*ΔHgb (g/dL) = SLS-Hgb - Corrected Hgb


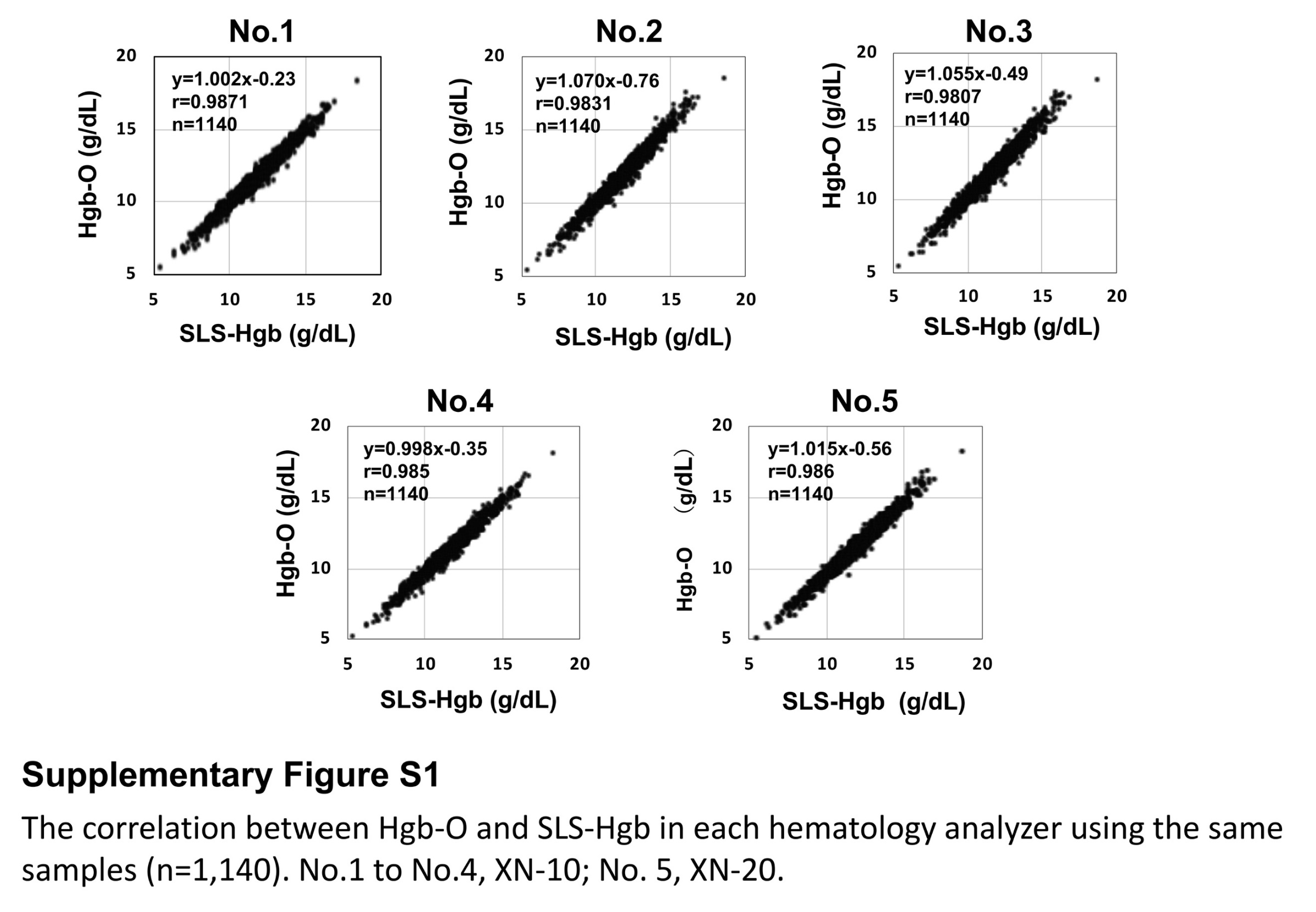

Supplement: Supplementary file 1 — Supplementary Information. [file 41598_2021_94394_MOESM1_ESM.docx]
